# Do acute elevations of serum creatinine in primary care engender an increased mortality risk?

**DOI:** 10.1186/1471-2369-15-206

**Published:** 2014-12-22

**Authors:** Helen Hobbs, Paul Bassett, Toby Wheeler, Michael Bedford, Jean Irving, Paul E Stevens, Christopher KT Farmer

**Affiliations:** East Kent University Hospitals NHS Foundation Trust, Kidney Kent Research Group, Kent Kidney Care Centre, Kent and Canterbury Hospital, Ethelbert Road, Canterbury, Kent CT1 3NG England; Statsconsultancy Ltd Amersham, Buckinghamshire, HP7 9EN UK

**Keywords:** AKI, Primary care, Mortality and epidemiology

## Abstract

**Background:**

The significant impact Acute Kidney Injury (AKI) has on patient morbidity and mortality emphasizes the need for early recognition and effective treatment. AKI presenting to or occurring during hospitalisation has been widely studied but little is known about the incidence and outcomes of patients experiencing acute elevations in serum creatinine in the primary care setting where people are not subsequently admitted to hospital. The aim of this study was to define this incidence and explore its impact on mortality.

**Methods:**

The study cohort was identified by using hospital data bases over a six month period.

Inclusion criteria: People with a serum creatinine request during the study period, 18 or over and not on renal replacement therapy.

The patients were stratified by a rise in serum creatinine corresponding to the Acute Kidney Injury Network (AKIN) criteria for comparison purposes. Descriptive and survival data were then analysed.

Ethical approval was granted from National Research Ethics Service (NRES) Committee South East Coast and from the National Information Governance Board.

**Results:**

The total study population was 61,432. 57,300 subjects with ‘no AKI’, mean age 64.The number (mean age) of acute serum creatinine rises overall were, ‘AKI 1’ 3,798 (72), ‘AKI 2’ 232 (73), and ‘AKI 3’ 102 (68) which equates to an overall incidence of 14,192 pmp/year (adult). Unadjusted 30 day survival was 99.9% in subjects with ‘no AKI’, compared to 98.6%, 90.1% and 82.3% in those with ‘AKI 1’, ‘AKI 2’ and ‘AKI 3’ respectively. After multivariable analysis adjusting for age, gender, baseline kidney function and co-morbidity the odds ratio of 30 day mortality was 5.3 (95% CI 3.6, 7.7), 36.8 (95% CI 21.6, 62.7) and 123 (95% CI 64.8, 235) respectively, compared to those without acute serum creatinine rises as defined.

**Conclusions:**

People who develop acute elevations of serum creatinine in primary care without being admitted to hospital have significantly worse outcomes than those with stable kidney function.

## Background

The prevalence of AKI is increasing [1, 2] associated with an aging population, the increase in comorbidities including obesity, diabetes and hypertension in the developed world and advances in medical practice including aggressive investigative procedures, invasive clinical procedures and increased sepsis [3, 4]. AKI is a complex disorder with multiple aetiologies and risk factors and may often be preventable and reversible. Clinically, its manifestations may range from a small isolated elevation in serum creatinine (SCr) to loss of urine output, advanced kidney failure, hyperkalaemia, disturbed whole body acid–base balance manifest as acidosis and fluid overload [4–12].

There is now evidence that even small isolated increases in serum creatinine have an associated increase in short-term morbidity and mortality, in longer-term outcomes including one year mortality [5, 13] and even more so when renal replacement therapy is required [1, 14–16].

The risk of AKI and the significant impact it has on patient morbidity and mortality emphasizes the need for early recognition and effective treatment. A concept highlighted in published clinical guidance [17] and the 2009 National Confidential Enquiry into Patient Outcome and Death in the setting of AKI in hospital, which uncovered systematic failings in the identification and subsequent management [2].

Although AKI presenting to or occurring during hospitalisation has been widely studied and reported in the literature, however all that data relates to in-patient studies. Little is known about the incidence and outcomes of patients experiencing acute elevations in SCr in primary care who are not subsequently admitted to hospital.

The hypothesis for the study was that acute elevations in SCr measured in the community may represent episodes of AKI and therefore will be associated with increased mortality in the community compared to those who did not have acute elevations in the serum creatinine. The aim of this study was to describe the incidence in the community of acute elevations in SCr defined using the Acute Kidney Injury Network (AKIN) criteria and the associated mortality in patients who had not been subsequently admitted to hospital. We aligned these acute changes to the AKIN criteria in order to compare outcomes with the published literature.

## Methods

This was an observational database study using data from two databases to collate the study cohort;

- The pathology database: East Kent is served by 3 acute hospitals with a single laboratory service feeding into a regional pathology database which holds all records of blood tests requested from GPs, outpatients and inpatients
- The hospital data warehouse: This contains data from coded episodes on all admissions and outpatient activity in East Kent.

Ethical approval was granted by the National Research Ethics Service (NRES) Committee South East Coast. National Information Governance Board (NIGB) approval was given to link the data on these databases.

The study cohort was recruited between 1st of February 2009 – 31st July 2009 from the East Kent population in the United Kingdom, numbering approximately 744,400 people of whom 582,300 were over the age of 18. This cohort was followed up either until the primary endpoint (death) or for a period of two years. All patients with a SCr request from the community, with a valid NHS number and aged 18 or over during the recruitment period were identified using the pathology database. The data was then linked to the hospital information system and pathology databases using the NHS number to ascertain outcomes and co-morbidity data. Patients were excluded from the data set if they had a non-elective admission to hospital during either the recruitment period or the follow up period, if they did not have a baseline SCr within 12 months of the incident SCr or that they were already on renal replacement therapy. The data was then pseudonymised in accordance with the NHS information governance standards to protect patient confidentiality [18].

**Figure 1:**
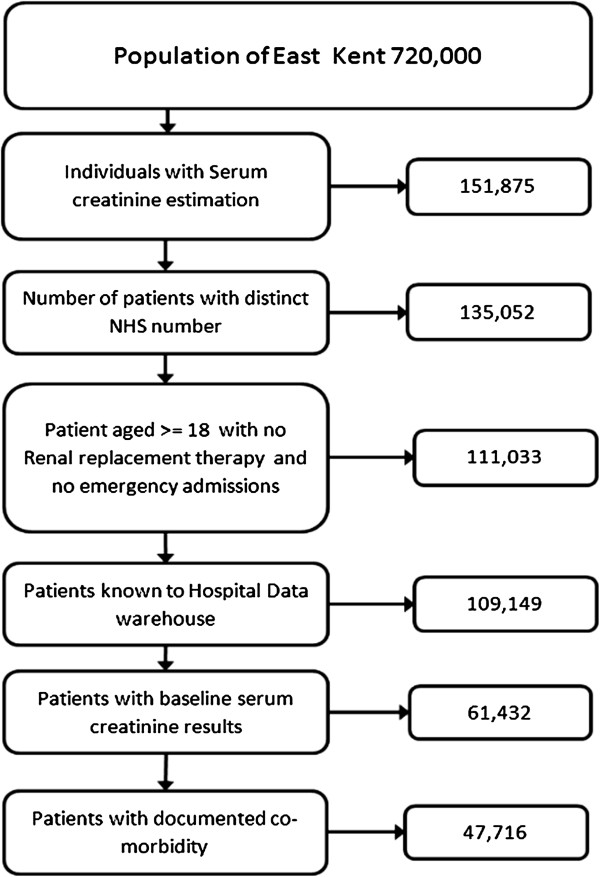
**Derivation of the study population.** This diagram shows the derivation of the study population with the numbers of patients remaining at each level exclusion criteria.

There were 135,052 patients with both unique NHS number and a SCr request from primary care identified between 1st of February 2009 and 31st July 2009, the study recruitment period; 24,019 people were then excluded because they were either under the age of 18, or they had an emergency admission during either the study recruitment period they were already on RRT, or the 24 months follow up period. The remaining 111,033 patients were identified using the NHS number on the hospital data warehouse to ascertain date of death and co-morbidity. Of these, 61,443 had a valid SCr that could be used as a baseline. A further 11 patients had to be removed as the data could not be validated, leaving the study population as 61,432. This process is shown in a data flow diagram (Figure 1). The patient cohort was then split into two study groups; people with and without acute elevations in SCr. Those with acute elevations were then defined and stratified according to the Acute Kidney Injury Network SCr criteria [19]. People who had emergency (non-elective admissions) during the study period were excluded and formed a cohort for a separate study [20].

The AKIN criteria require a baseline SCr within 48 hours of the index value, however in primary care blood tests are seldom repeated this frequently, for that reason the lowest SCr in the twelve months prior to the index value was used as the baseline. This method has been previously described by LaFrance and Miller [21]. If an individual with a raised SCr had multiple blood tests during the study recruitment period the highest SCr was used. In those people not fulfilling AKIN criteria the date of the first SCr in the recruitment period was used as the incident creatinine for subsequent follow up. For descriptive purposes those not fulfilling AKIN criteria were referred to as “no AKI”, and those fulfilling AKIN criteria as “AKI 1”, “AKI 2” and “AKI 3” (Table 1).

**Table 1:** **Definitions for the stage of AKI: using the AKI network criteria**

Stage	Serum creatinine criteria
Stage 1	1.5 – 1.9 times baseline creatinine OR an increase in serum creatinine of > 0.3 mg/dl (>26.5mmols/l*)
Stage 2	2.0 – 2.9 times baseline creatinine 0.5 ml/kg/h for X12 hours
Stage 3	3.0 times the baseline OR Increase in serum creatinine to >4.0 mg /dl (353.6 mmols/l**)

This table shows the Acute Kidney Injury Network serum creatinine criteria, which was used to stratify the primary care cohort. *AKI defined only using these criteria has been referred to as AKI 1a in this paper. **AKI defined only using these criteria had been referred to as AKI 3a.^19^

Charlson co-morbidity scores were used to measure disease burden and was calculated according to the method developed by the Dr Foster Unit, published by Bottle and Aylin [22].

Serum creatinine was measured using an enzymatic method on Abbott Architect analysers (Abbott Diagnostics, Maidenhead, Berkshire, UK). The enzymatic method for creatinine is standardized against NIST SRM 967 and thus is traceable to isotope dilution mass spectrometry (ID-MS). The assay was related to an ID-MS assay according to the equation: Abbott enzymatic = 0.982 (ID-MS) + 3.3 (n = 203). The assay is related to the Roche creatinine plus enzymatic assay (Hoffman-La Roche, Basel, Switzerland) used to re-express the MDRD equation according to the equation: Abbott enzymatic = 1.0338 (Roche enzymatic) + 0.98, unpublished data, E.J. Lamb). In order to establish baseline kidney function in all patients Glomerular Filtration Rate (GFR) was calculated using the Modified Diet in Renal Disease (MDRD) formula [23].

The association between acute elevations in SCr categorised by AKIN criteria and survival was examined. The primary outcome assessed was all cause mortality. The results were expressed as Kaplan-Meier survival curves, first unadjusted and then adjusted for age, gender, stage of Chronic Kidney Disease (CKD) and Charlson co-morbidity score. Cox regression survival analysis was then used to examine the temporal relationship between the incident creatinine and death. These results were expressed as hazard ratios.

Finally the probability of dying at 30 days and12 months was estimated using logistic regression analysis. To allow for possible confounding variables, a series of four models were fitted with varying adjustments for other variables to allow comparison between the categories

The models fitted were as follows:

1. Unadjusted relationship between elevated serum creatinine defined by AKIN criteria and death
2. The relationship between elevated serum creatinine defined by AKIN criteria and death, adjusted for age and sex only
3. The relationship between elevated serum creatinine defined by AKIN criteria and death, adjusted for age, sex, baseline CKD stage & using individual co-morbidities
4. The relationship between elevated serum creatinine defined by AKIN criteria and death,, for age, sex, baseline CKD stage & Charlson score

There was a non-linear relationship between age and all outcomes, therefore a linear and squared term were included for age to best fit the observed relationships.

Around 22% of patients had missing comorbidity data, and thus potentially excluded from models 3 and 4. In order to retain these patients in the analysis, an additional 'missing' category was included for the Charlson score in model 4. Each of the individual comorbidities was missing for the same patients, and thus it was not possible to include an additional category for each of these. Instead it was assumed that the individual comorbidities were absent for those with missing data, and an additional variable, missing comorbidity or not, was included in the model. Since none of the patients with AIDS died within 30 days of the incident creatinine AIDS was omitted from the analysis of 30-day mortality.

The data was then re-analysed dividing patient s into 6 groups based on their AKI status, no AKI, AKIN1, AKIN1a, AKIN2, AKIN3 and AKIN3a. The aim of the all analyses was to compare between the six AKI groups. An additional variable was added to the model three and four to adjust for time between incident creatinine and baseline creatinine (models 5, 6, 7 & 8).

## Results

There were 61,432 people were included in the study cohort. The mean ages of people with no AKI, AKI 1, AKI 2 and AKI 3 were 64.1 years, 72.2 years, 72.9 years and 67.7 years respectively. The gender split shows females as slightly under represented in AKI 1 and AKI 3 and slightly over represented in groups “no AKI” and AKI 2.

Approximately 7% (4,132/61,432) of people had an episode of AKI in the study recruitment period, the total number of patients in each category were, AKI 1 (3,798), AKI 2 (232), AKI 3 (102) (Table 2).

**Table 2:** **Patient characteristics for all patients studied**

Variable	All (n = 61,432)	No AKI (n = 57,300)	AKI 1 (n = 3,798)	AKI 2 (n = 232)	AKI 3 (n = 102)	P-value ^(*)^
Age in years mean (SD)	64.1 (15.8)	64.1 (15.8)	72.2 (14.4)	72.9 (13.3)	67.7 (15.4)	<0.001
−39 (%)	7.8	8.4	4.0	2.2	7.8	<0.001
40-59 (%)	24.9	26.4	12.4	12.5	17.7	
60-79 (%)	49.4	49.4	51.3	51.3	50.0	
80 + (%)	17.9	15.8	32.4	34.1	24.5	
Female (%)	55.1	55.7	46.2	58.6	43.1	<0.001
Baseline GFR						
>60 (%)	77.4	79.2	52.0	71.1	51.0	<0.001
45 - <60 (%)	15.4	15.0	20.8	18.1	7.8	
30 - <45 (%)	5.6	4.8	17.1	7.8	6.9	
<30 (%)	1.6	1.0	10.1	3.0	34.3	

This table shows age at incident creatinine, gender and baseline GFR. (*)All comparisons performed using the Chi-square test, except for age on a continuous scale, where analysis of variance (ANOVA) was used.

We subsequently analysed the data using both MDRD and CKD–EPI estimating equations, we only present the results using MDRD as the frequency distribution using CKD-EPI was similar and the former is the more commonly used. People with a GFR of less than 60 were more likely to have an episode of AKI. In patients with stage 4 or 5 CKD only 7% of patients with AKI 1 and 3% of AKI 3 were defined using a relative change in creatinine. Despite the likelihood of inherent variability in SCr estimation contributing to elevations in SCr in those with more advanced CKD only 10% of those with AKI 1 had stage 4 and 5 CKD (Table 2).

Co-morbidity data were available in 78% of the study cohort. Patients fulfilling AKIN criteria had more co-morbidity than those who did not. In general this was associated with increasing elevation in SCr shown in Table 3. The association between SCr elevation and Charlson co-morbidity score was examined as a categorical variable. The proportion of patients with a Charlson co-morbidity score of ≤0 fell with increasing SCr elevation, from 67% of patients with “no AKI” to 22% with AKI 3 (Table 3).

**Table 3:** **Prevalence of comorbidities in the study cohort**

Variable	Total (47,716)	No AKI (n = 43,960)	AKI 1 (n = 3,442)	AKI 2 (n = 223)	AKI 3 (n = 91)	P-value ^(*)^
Co - morbidity						
AIDS (%)	0.1	0.1	0.1	0.0	0.0	0.71
Malignancy** (%)	6.7	6.0	14.2	19.7	18.7	<0.001
CHF (%(%)	3.0	2.2	11.8	19.3	4.4	<0.001
COPD (%)	9.1	8.6	15.2	15.7	11.0	<0.001
Stroke/TIA (%)	4.3	3.9	9.0	12.6	6.6	<0.001
Dementia (%)	0.8	0.7	2.1	2.7	0.0	<0.001
Diabetes (%)	11.8	10.9	22.3	24.2	24.2	<0.001
Hemiplegia (%)	1.0	1.0	1.5	1.8	2.2	0.01
Hypertension (%)	28.0	26.2	48.6	56.1	55.0	<0.001
MI (%)	4.1	3.7	8.5	10.3	4.4	<0.001
Solid tumour** (%)	1.1	0.9	2.5	7.2	4.4	<0.001
Liver disease (%)	1.0	0.9	2.1	4.9	0.0	<0.001
PVD (%)	2.3	1.9	7.0	6.3	8.8	<0.001
Peptic ulcer (%)	0.6	0.5	1.5	2.2	1.1	<0.001
CKD (%)	3.1	1.9	16.9	14.8	37.4	<0.001
Rheumatic dis. (%)	3.4	3.1	6.6	9.4	7.7	<0.001
Charlson score:						
≤0	64.3	66.9	34.1	28.3	22.0	<0.001
1-10	23.5	22.8	31.2	24.7	38.5	
11+	12.3	10.3	34.7	47.1	39.6	

(*)P-values comparing difference between four AKI categories.

(**)These two variables are mutually exclusive because ‘Solid tumour’ may contain benign tumours.

This table shows the proportions of comorbidities for people who had comorbidity recorded (n = 47,716). All comparisons performed using the Chi-square test.

Chronic Heart Failure (CHF), Chronic Obstructive Pulmonary Disease (COPD), Myocardial Infarction (MI), Peripheral Vascular Disease (PVD), Chronic Kidney Disease (CKD).

**Figure 2:**
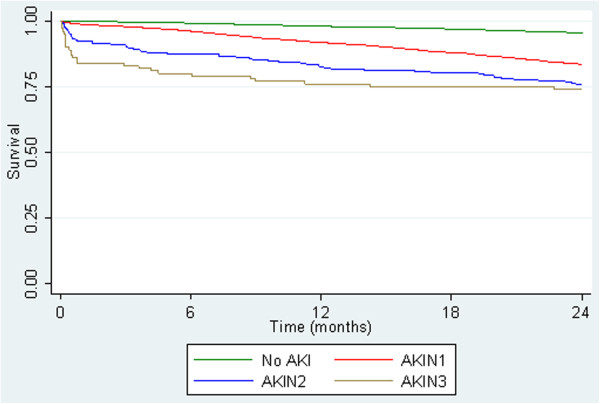
**Unadjusted Kaplan-Meier survival curves by AKIN criteria.** This figure shows the survival curves by AKIN criteria up to 24 months post incident serum creatinine using unadjusted data. All patients (n = 61,432). The majority of the effect is seen in the first 30 days.

There was a total of 3,301 deaths from 61,432 patients, 2582 (4.5%) with ‘No AKI’, 630 (16.6%), AKI 1 61 (26.3%) AKI 2 and 28 (27.5%) AKI 3. Survival analysis using unadjusted data demonstrated that those who fulfilled AKIN criteria did worse than those who did not with 98% of patients still alive at 12 months compared with 92% AKI 1, 83% AKI 2, and 76% AKI 3 (Table 4). The majority of this effect was seen in the first thirty days and the probability of survival at 30 days fell 99% in AKI 1 to 84% in AKI 3 (Figure 2).

**Figure 3:**
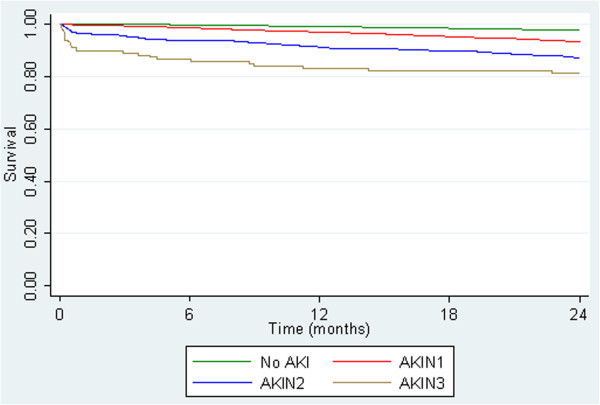
**Adjusted Kaplan-Meier survival curves by AKIN criteria.** This figure shows the survival curves by AKIN criteria up to 24 months. Adjusted for age at incident creatinine, gender, baseline GFR and Charlson score. Patients with co-morbidity data (n = 47,716).

This effect was still apparent when adjusted for age gender, co-morbidity and baseline GFR (Figure 3).

**Table 4:** **A summary of the survival estimates**

Variable	No AKI	AKIN 1	AKIN 2	AKIN 3
Number of patients	57,300	3,798	232	102
Number of deaths	2,582	630	61	28
% of deaths	4.5	16.6	26.3	27.5
1 m survival (95% CI)	0.999 (0.999, 0.999)	0.987 (0.983, 0.990)	0.925 (0.882, 0.953)	0.840 (0.752, 0.899)
6 m survival (95% CI)	0.992 (0.991, 0.992)	0.961 (0.954, 0.967)	0.872 (0.967, 0.970)	0.800 (0.707, 0.866)
12 m survival (95% CI)	0.980 (0.979, 0.981)	0.918 (0.909, 0.926)	0.827 (0.772, 0.871)	0.760 (0.664, 0.824)
18 m survival (95% CI)	0.969 (0.967, 0.970)	0.880 (0.869, 0.890)	0.801 (0.743, 0.847)	0.750 (0.653, 0.824)
24 m survival (95% CI)	0.955 (0.953, 0.957)	0.835 (0.823, 0.846)	0.757 (0.695, 0.807)	0.740 (0.642, 0.815)

This table shows the survival estimates for the 2 year follow up period along with corresponding confidence intervals in 6 monthly intervals.

Cox regression analysis shows that the increased risk of death at 24 months as, 1.81 (1.65, 1.99), 2.76 (2.11, 3.62), 2.76 (2.11, 3.62) respectively compared to the reference range (p < 0.001). This suggests a temporal relationship between acute elevation in SCr and mortality (Table 5).

**Table 5:** **This table shows Logistic regression analysis for 30 day survival**

Model	Variable		Odds ratio (95% CI)	P-value
1	-No AKI		1	<0.001
	-AKIN 1		11.4 (7.98, 16.3)	
	-AKIN 2		88.7 (54.4, 145)	
	-AKIN 3		173 (98.7, 302)	
2	-No AKI		1	<0.001
	-AKIN 1		6.74 (4.67, 9.72)	
	-AKIN 2		58.9 (35.4, 98.0)	
	-AKIN 3		158 (87.0, 288)	
	Female sex		0.69 (0.50, 0.95)	<0.001
	Age ^(*)^	-Linear term	0.61 (0.22, 1.67)	<0.001
		-Squared term	1.09 (1.02, 1.18)	
3	-No AKI		1	<0.001
	-AKIN 1		5.42 (3.67, 8.00)	
	-AKIN 2		39.8 (22.8, 69.6)	
	-AKIN 3		185 (94.0, 365)	
	Female sex		0.67 (0.48, 0.94)	0.02
	Age ^(*)^	-Linear term	0.39 (0.14, 1.10)	<0.001
		-Squared term	1.13 (1.06, 1.22)	
	Any malignancy		3.68 (2.36, 5.74)	<0.001
	CHF		2.16 (1.29, 3.65)	0.004
	CPD		1.00 (0.59, 1.69)	1.00
	Cerebrovascular disease		0.54 (0.27, 1.08)	0.08
	Dementia		4.87 (2.50, 9.47)	<0.001
	Diabetes		1.21 (0.77, 1.89)	0.42
	Hemiplegia or paraplegia		2.94 (0.96, 9.01)	0.06
	Hypertension		1.14 (0.79, 1.66)	0.48
	MI		0.82 (0.41, 1.64)	0.57
	Solid tumour		7.55 (4.20, 13.6)	<0.001
	Mild liver disease		2.28 (0.83, 6.32)	0.11
	PVD		1.21 (0.60, 2.43)	0.60
	Peptic ulcer		2.89 (1.05, 7.98)	0.04
	Renal disease		0.46 (0.23, 0.91)	0.03
	Rheumatic disease		0.56 (0.19, 1.59)	0.28
	Missing data		1.52 (0.87, 2.67)	0.14
	GFR > 60		1	0.78
	GFR 45 - < 60		0.82 (0.53, 1.26)	
	GFR 30 - < 45		0.83 (0.49, 1.42)	
	GFR < 30		0.85 (0.38, 1.89)	
4	-No AKI		1	<0.001
	-AKIN 1		5.26 (3.57, 7.74)	
	-AKIN 2		36.8 (21.6, 62.7)	
	-AKIN 3		123 (64.8, 235)	
	Female sex		0.74 (0.53, 1.02)	0.07
	Age ^(*)^	-Linear term	0.52 (0.19, 1.42)	<0.001
		-Squared term	1.10 (1.03, 1.18)	
	Charlson	-≤0	1	<0.001
		-1-10	2.67 (1.59, 4.49)	
		-11+	5.24 (3.25, 8.44)	
		-No info	2.05 (1.11, 3.79)	
	GFR > 60		1	0.03
	GFR 45 - < 60		0.65 (0.43, 1.00)	
	GFR 30 - < 45		0.63 (0.38, 1.06)	
	GFR < 30		0.42 (0.20, 0.92)	

(*)Odds Ratio reported for a 10-year increase in age.

This table shows the logistic regression analysis for the like-hood of dying in the first 30 days following an acute elevation in serum creatinine. Model 2 adjusts for age and gender, model 3 also adjusts for individual co-morbidity and model 4 replaces co-morbidity with a Charlson score to measure the effect of cumulative co-morbidity burden.

Chronic Heart Failure (CHF), Chronic Obstructive Pulmonary Disease (COPD), Myocardial Infarction (MI), Peripheral Vascular Disease (PVD), Chronic Kidney Disease (CKD).

Logistic regression analysis for all-cause mortality at 30 days suggested a highly significant difference between AKI groups in all models, showing that patients with a higher stage of AKI had an increased likelihood of death. The unadjusted analyses indicated that likelihood of death in AKI 1 patients increased by 11.4 times that of patients with no AKI, increasing to a 180 time more likely for patients with AKI 3. The impact AKI had on likelihood of death was attenuated after adjusting for some confounding variables. When adjusting for disease burden in model 4, death in the first 30 days was, 5.26, 36.8 and 123 times more likely compared to those with ‘No AKI’ (Table 6).

**Table 6:** **This table shows logistic regression analysis at 30 days with AKI defined by absolute change as separate groups**

Model	Variable		Odds Ratio (95% CI)	P-value
5	No AKI		1	<0.001
	-AKI 1		18.8 (12.0, 29.7)	
	-AKI 1a		8.26 (54.4, 12.9)	
	-AKI 2		88.7 (98.7, 145)	
	-AKI 3		269 (148, 488)	
	-AKI 3a		24.4 (3.30, 181)	
6	No AKI		1	<0.001
	-AKI 1		14.0 (8.84, 22.2)	
	-AKI 1a		4.42 (2.81, 6.97)	
	-AKI 2		59.4 (35.7, 98.8)	
	-AKI 3		233 (121, 447)	
	-AKI 3a		25.6 (3.33, 197)	
	Female sex		0.61 (0.44, 0.85)	<0.001
	Age^(*)^	-Linear term	0.61 (0.22, 1.68)	<0.001
		-Squared term	1.09 (1.02, 1.17)	
7	No AKI		1	<0.001
	-AKI 1		9.69 (5.91, 15.9)	
	-AKI 1a		3.99 (2.46, 6.49)	
	-AKI 2		42.9 (24.5, 75.1)	
	-AKI 3		237 (115, 490)	
	-AKI 3a		44.5 (4.83, 409)	
	Female sex		0.62 (0.44, 0.88)	0.007
	Age^(*)^	-Linear term	0.39 (0.14, 1.10)	<0.001
		-Squared term	1.13 (1.06, 1.22)	
	Any malignancy		3.62 (2.31, 5.67)	<0.001
	CHF		2.32 (1.36, 3.94)	0.002
	CPD		1.07 (0.63, 1.82)	0.81
	Cerebrovascular disease		0.48 (0.23, 0.98)	0.05
	Dementia		4.88 (2.48, 9.60)	<0.001
	Diabetes		1.19 (0.75, 1.89)	0.45
	Hemiplegia or paraplegia		2.87 (0.91, 9.09)	0.07
	Hypertension		1.24 (0.85, 1.80)	0.26
	MI		0.79 (0.39, 1.62)	0.52
	Solid tumour		8.15 (4.53, 14.6)	<0.001
	Mild liver disease		2.48 (0.88, 7.01)	0.09
	PVD		1.18 (0.58, 2.41)	0.64
	Peptic ulcer		2.82 (1.00, 7.93)	0.05
	Renal disease		0.48 (0.24, 0.96)	0.04
	Rheumatic disease		0.63 (0.22, 1.81)	0.39
	Missing data		1.45 (0.82, 2.54)	0.20
	GFR > 60		1	0.92
	GFR 45 - < 60		0.95 (0.61, 1.48)	
	GFR 30 - < 45		0.92 (0.53, 1.58)	
	GFR < 30		1.24 (0.53, 2.91)	
	Time measurement ^(†)^		0.84 (0.80, 0.89)	<0.001
8	No AKI		1	<0.001
	-AKI 1		9.55 (5.89, 15.5)	
	-AKI 1a		3.63 (2.24, 5.87)	
	-AKI 2		38.9 (22.8, 66.3)	
	-AKI 3		163 (81.6, 327)	
	-AKI 3a		27.0 (3.00, 243)	
	Female sex		0.67 (0.48, 0.94)	0.02
	Age ^(*)^	Linear term	0.54 (0.20, 1.48)	<0.001
		Squared term	1.10 (1.03, 1.18)	
	Charlson	≤0	1	<0.001
		1-10	2.92 (1.73, 4.93)	
		11+	5.95 (3.68, 9.64)	
		No info	1.97 (1.06, 3.66)	
	GFR > 60		1	0.45
	GFR 45 - < 60		0.80 (0.52, 1.23)	
	GFR 30 - < 45		0.70 (0.41, 1.18)	
	GFR < 30		0.69 (0.31, 0.56)	
	Time measurement ^(†)^		0.84 (0.80, 0.88)	<0.001

(*) Odds Ratio reported for a 10-year increase in age.

(†) Time from baseline to AKI creatinine measurement (months).

This table shows the logistic regression analysis for the like-hood of dying in the first 30 days following an acute elevation in serum creatinine. Model 6 adjusts for age and gender, model 7 also adjusts for individual co-morbidity and time between incident creatinine and baseline creatinine, model 8 replaces co-morbidity with a Charlson score to measure the effect of cumulative co-morbidity burden and time between incident creatinine and baseline creatinine.

Chronic Heart Failure (CHF), Chronic Obstructive Pulmonary Disease (COPD), Myocardial Infarction (MI), Peripheral Vascular Disease (PVD), Chronic Kidney Disease (CKD).

Similar analyses were performed at 12 months (data not shown). This showed a similar but less marked effect with increasing follow up.

**Figure 4:**
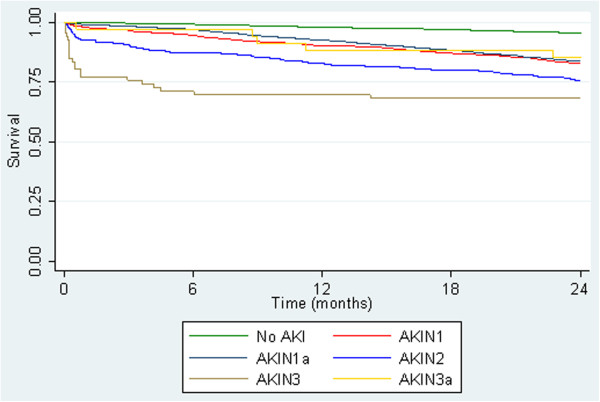
**Kaplan-Meier Survival by AKIN Criteria, including AKI defined by absolute change alone, all patients (n = 61,432).** This figure shows the survival criteria up to 24 months this is split into two categories to show the effect on those defined using and an absolute change only compared against those defined using a relative change.

However the size of the effect was altered when accounting for differences between those who were defined using an absolute or relative change in AKI 1 and 3 (Figure 4).

Those defined by an absolute change having better survival than those defined by a relative change in serum creatinine. Cox survival analysis at 30 days showed AKI 1a 0.991 (0.983, 0.99) AKI 1 0.979 (0.969, 0.986), AKI 2 0.925 (0.882, 0.953), AKI 3a 0.971 (0.809, 0.996), AKI 3 0.773 (0.652, 0.856)

Logistic regression analysis for all-cause mortality suggested a highly significant association between AKI groups and death within 30 days, with the general trend being that a higher AKI stage was associated with an increased risk of an early death. After adjusting for the individual comorbidities (model 7), the odds of 30 day mortality for AKI 1a patients was 4 times greater than for patients with no AKI, and 10 times time for AKI 1 patients. The odds were over 40 times greater for AKI 2 and AKI 3a patients than for those with no AKI, whilst the odds were over 200 times greater for AKI 3 patients, however the Confidence Intervals (CI) were large and the numbers in AKI 3a and AKI 3 (Table 7).

**Table 7:** **Number of incidences of AKI for each Stage of CKD**

		Stage CKD (MDRD)					Total
		Stage 1	Stage 3a	Stage 3b	Stage 4	Stage 5	
AKI Stage with absolutes	No AKI	45377	8612	2736	552	23	57300
	AKI 1	939	90	81	27	1	1138
	AKI 1a*	1037	698	568	339	18	2660
	AKI 2	165	42	18	7	0	232
	AKI 3	52	8	7	1	0	68
	AKI 3a**	0	0	0	3	31	34
Total		47570	9450	3410	929	73	61432

This table shows the number of incidences at each stage AKI per stage of CKD, using the AKIN Criteria. * Defined using an absolute change of >26.5mmols/l alone. ** Defined using an absolute change of > 353.6 mmols/l with an acute increase of at least 0.5 mg/dl (44μmols/l) alone.

## Discussion

This study is the first to describe the survival outcome associated with acute elevations in SCr occurring entirely in primary care without subsequent non-elective admission to hospital. However there have been a small number of in-hospital studies that have compared community acquired AKI ( CA-AKI) with Hospital acquired (HA-AKI) which have found that number of CA-AKI was higher than HA- AKI on a ratio 3:1 [24–26]. The incidence of acute elevations in SCr fulfilling AKIN criteria in our study population was estimated to be 14,132 per million adult population per year (pmap/yr) which is similar in comparison to a contemporaneous in-patient study of the same population, which estimated an in-hospital incidence of 15,352 pmap/yr [20]. This implies that in total 5% of the adult population of East Kent have experienced an acute elevation in SCr fulfilling AKIN criteria during the study time period. Although the majority of the acute elevations in SCr in managed in primary care were skewed to AKI 1 than in other in-hospital studies [20].

We have been careful to describe these acute elevations in SCr as fulfilling AKIN creatinine criteria rather than defined as AKI because it was not possible to fulfil the time constraints for either the AKIN or RIFLE [10] definitions of AKI because blood tests in the community are seldom repeated within 48 hours. Instead we used the methodology described by La France and Miller^21^ which extends the time to reference creatinine to 12 months. Whilst this may increase the incidence of acute elevations in SCr corresponding to AKIN stage 1, relative to time to baseline creatinine, the evidence suggests that such small rises have a significant adverse effect on outcome in hospital inpatients [7]. However, in model 7 and 8 we adjusted the analysis for time to baseline in months. Despite the fact that this showed each months increase had a very small significant effect on reducing the risk of mortality, this is unlikely to have large impact on outcome (Table 7).

Our data supports the opinion that the risk of mortality is directly associated with acute elevation in serum creatinine occurring and managed entirely in primary care. This risk is still apparent even in small rises in serum creatinine. Although this may be associated with pre-existing higher levels of mortality compared with the reference group the risk of mortality in the first 30 days is significant. These results are comparable to the in-hospital study [20] which also reports and increasing risk of death by stage in 30 days. Whilst the community study had fewer patients with AKI 2 and AKI 3, this was offset by larger numbers of AKI 1 giving a similar overall incidence. Although there are no comparable studies in primary care, other studies in Wales and in the USA [24–26] comparing CA-AKI and HA-AKI have also found an association between AKI and survival in the first 30 days, and that the risk of death increases with stage AKI. These similarities suggest that acute elevations in serum creatinine occurring and managed in the community may represent a health risk and merit further investigation and better understanding. It will be important to ascertain why the tests were requested, whether these acute SCr elevations in primary care are actually recognised and prompt further action, or whether they go unrecognised by the healthcare system. This is something we have hypothesised may contribute to the development of CKD in the general population [27].

Furthermore this study showed an excess of AKI 1 and 3 in people with advanced CKD (Stage 4&5). The majority of this was defined by an absolute change in serum creatinine rather than a relative change (Table 5). When analysed separately those defined by an absolute change had less of a risk that those defined by a relative change. Although AKI 1a had a smaller risk than AKI 1 it was still significant when compared to the reference group. While the patient numbers are small, the survival rate of those in AKI 3a was significantly better than those in AKI 3. Since all those grouped in 3a had stage 4 or 5 CKD it may be possible that this group does not represent an acute episode and suggests that the AKIN criteria in advanced CKD may be overly sensitive. This has been shown in other studies [28].

This study has limitations, firstly patients were recruited from a single county in the United Kingdom. Whilst the study recruited large numbers of patients the population of East Kent, it is not entirely representative of the United Kingdom demographic therefore the population studied is older with fewer ethnic minorities than the national average [29]. However, it is not unreasonable to assume that primary care practice in Kent is similar to that in the rest of the UK. Never the less care should be taken when relating the findings to an international setting particularly bearing in mind that provision of primary care services differs considerably, particularly across Europe.

Secondly, as this was an observational study and patients could only be included if they had had a SCr test, therefore the study population may represent a more co-morbid population that the general population. Also co-morbidity data was taken from hospital records and only patients who had had hospital admissions prior to the study period would have recorded co-morbidity. Although Charlson Scores based on administrative data may underestimate the prevalence of co-morbid disease NHS administrative data accuracy has been shown to be improving in recent years. Systematic review suggests that current levels of reported accuracy are sufficiently robust to support their use for research [22, 30]. Although it is probable that some patients with significant co-morbidity had none recorded (either because of no previous admission or an incomplete record) it is worth noting that 47,716 of 61,432 patients included in the study had recorded co-morbidity. Furthermore the absence of a co-morbidity record was included in the multivariate model allowing the model to account for missing variables, particularly as they are not missing at random.

Finally, the study only examined the hospital records of the local NHS hospital Trust, it is possible that patients could have been admitted to other surrounding hospitals or non NHS providers. However, in reality very few patients are admitted to non NHS providers as non-elective patients in the UK. The recruitment area was a peninsula (East Kent, UK) the vast majority of acute admissions in the area are to the single provider examined.

This study group was a carefully selected cohort of patients with no acute hospital admissions in the eighteen months follow up period, thus removing the potential confounding of subsequent hospital admission. However, this may mean that we have underestimated the impact of acute elevations of SCr in primary care. It is also likely that patients with higher acute SCr elevations were recognised and admitted to hospital.

## Conclusion

In conclusion, this study suggests patients who develop acute SCr elevations in primary care and are not subsequently admitted to hospital have a significantly increased mortality compared to those whose SCr is stable. The increased mortality appears to be temporally associated with the acute SCr elevation, i.e. within 30 days, suggesting a direct association between the event and adverse outcome. This association persists even after multiple adjustments for potential confounders. Further studies are required to examine the potential causal association between these events in primary care and poor outcome. Clearly if there is a causal relationship this would be an important area in which to intervene.

## Abbreviations

AKI: Acute Kidney Injury
AKIN: Acute Kidney Injury Network
CA-AKI: Community Acquired Acute Kidney Injury
CI: Confidence Intervals
CKD: Chronic Kidney Disease
CKD: EPI - Chronic Kidney Disease EPI
GFR: Glomerular Filtration Rate
HA: AKI - Hospital Acquired Acute Kidney Injury
NIGB: National Information Governance Board
NRES: National Research Ethics Service
MDRD: the Modified Diet in Renal Disease
SCr: Serum creatinine.
